# Oncogenic CagA Promotes Gastric Cancer Risk via Activating ERK Signaling Pathways: A Nested Case-Control Study

**DOI:** 10.1371/journal.pone.0021155

**Published:** 2011-06-16

**Authors:** Jae Jeong Yang, Lisa Y. Cho, Seung Hyun Ma, Kwang-Pil Ko, Aesun Shin, Bo Youl Choi, Dong Soo Han, Kyu Sang Song, Yong Sung Kim, Soung-Hoon Chang, Hai-Rim Shin, Daehee Kang, Keun-Young Yoo, Sue K. Park

**Affiliations:** 1 Department of Preventive Medicine, Seoul National University College of Medicine, Seoul, Korea; 2 Department of Preventive Medicine, Gachon University of Medicine and Science, Incheon, Korea; 3 Cancer Epidemiology Branch, National Cancer Center, Goyang-si, Korea; 4 Department of Preventive Medicine, Hanyang University College of Medicine, Seoul, Korea; 5 Department of Internal Medicine, Hanyang University College of Medicine, Seoul, Korea; 6 Department of Pathology, Chungnam National University College of Medicine, Daejeon, Korea; 7 Medical Genomics Research Center, Korea Research Institute of Bioscience and Biotechnology, Daejeon, Korea; 8 Department of Preventive Medicine, Konkuk University, Chungju, Korea; 9 Non Communicable Diseases and Health Promotion, World Health Organization, Western Pacific Regional Office, Manila, Philippines; 10 Department of Biomedical Science, Seoul National University Graduate School, Seoul, Korea; 11 Cancer Research Institute, Seoul National University, Seoul, Korea; Duke-National University of Singapore Graduate Medical School, Singapore

## Abstract

**Background:**

CagA cellular interaction via activation of the ERK signaling pathway may be a starting point in the development of gastric cancer. This study aimed to evaluate whether genes involved in ERK downstream signaling pathways activated by CagA are susceptible genetic markers for gastric cancer.

**Methods:**

In the discovery phase, a total of 580 SNPs within +/−5 kbp of 30 candidate genes were genotyped to examine an association with gastric cancer risk in the Korean Multi-center Cancer Cohort (100 incident gastric cancer case-control sets). The most significant SNPs (raw or permutated *p* value<0.02) identified in the discovery analysis were re-evaluated in the extension phase using unconditional logistic regression model (400 gastric cancer case-control sets). Combined analyses including pooled- and meta-analysis were conducted to summarize all the results.

**Results:**

24 SNPs in eight genes (ERK, Dock180, C3G, Rap1, Src, CrkL, Mek and Crk) were significantly associated with gastric cancer risk in the individual SNP analyses in the discovery phase (*p*<0.05). In the extension analyses, ERK rs5999749, Dock180 rs4635002 and C3G rs7853122 showed marginally significant gene-dose effects for gastric cancer. Consistently, final combined analysis presented the SNPs as significantly associated with gastric cancer risk (OR = 1.56, [95% CI: 1.19–2.06], OR = 0.61, [95% CI: 0.43–0.87], OR = 0.59, [95% CI: 0.54–0.76], respectively).

**Conclusions:**

Our findings suggest that ERK rs5999749, Dock180 rs4635002 and C3G rs7853122 are genetic determinants in gastric carcinogenesis.

## Introduction

Helicobacter pylori (*H. pylori*) is a proven cause of gastric carcinogenesis [1]. Nevertheless, its absolute effect can be modified by individual susceptibility risk factors such as genetic variants and *H. pylori* virulent characteristics [2], [3]. Cytotoxin-associated antigen (CagA), a *H. pylori* immunoprotein, is a crucial factor for individual susceptibility and is associated with severe clinical outcomes including gastric cancer [4], [5], [6]. In gastric epithelial cells, CagA interferes with diverse signal transduction pathways, such as Dock180-Rac1-WAVE-Arp2/3, C3G-Rap1-BRaf-MEK, Sos1-HRas-Raf1, and Ras-Raf-MEK-ERK signaling [3], [7], [8], [9]. Signals modified by CagA induce cellular changes such as apoptosis, proliferation, and cell mortality, and stimulate gastric carcinogenesis [10], [11], [12].

Among several downstream pathways activated by CagA, the extracellular signal-regulated kinase (ERK) cascade is a core pathway as it plays an important role in gastric carcinogenesis. CagA cellular interactions with Src, SHP2, Crk, CrkL or GRB2 are significantly associated with ERK activation, and other diverse proteins involved in CagA signaling are intimately connected to ERK signal pathways [10], [11], [12], [13]. Proteins can be regulated by their host genes; therefore, genes encoding proteins related to CagA and ERK signaling process may be important for gastric carcinogenesis but few studies have focused on these genetic polymorphisms.

CagA oncogenicity may be a starting point in the development of gastric cancer via activation of the ERK signal pathway. These signal transductions appear to be modified by host genetic variants. Thus, we hypothesized that genes involved in the ERK downstream signaling pathways activated by CagA may be susceptible genetic markers for gastric cancer. To evaluate this hypothesis, we conducted a multi-stage genetic association study that included 1) discovery phase: a candidate gene analysis that focused on 30 genes, Crk, CrkL, Csk, GRB2, c-Met, NFATC4, PTPN11, SMS, SOS1, Src, ERK, FAK, PLCγ, KRAS, NRAS, BRAF, RAF1, MAP2K1, MAP2K3, MAP2K4, MAP2K5, MAP2K6, p21, Dock180, RAC1,RAP1, WAVE, Arp2, Arp3 and C3G, involved in the CagA and ERK signal transduction pathway, and 2) extension phase that examined the most significant SNPs identified in the discovery analysis.

## Methods

### Study population

#### Discovery phase

The discovery candidate gene analysis was a population-based nested case-control study within the Korean Multi-Center Cancer Cohort (KMCC). From 1993 to 2004, the KMCC recruited a total of 19,688 participants from four urban and rural areas in Korea. All participants completed detailed standardized questionnaires by personal interview after informed consent. Blood and urine samples were also collected. Through record linkages to the national death certificate, the health insurance medical records databases and the national cancer registry, all participants were passively followed-up, and newly diagnosed cases were ascertained. Detailed information about the KMCC is described elsewhere [14]. In December of 2005, 249 gastric cancer cases defined according to the International Statistical Classification of Diseases and Related Health Problems 10th Revision (ICD-10, C16) were identified. Cases diagnosed before recruitment (n = 52), with no blood samples (n = 35), or insufficient DNA level under 50 ng/µl (n = 62) were excluded. Cancer-free controls were matched by age (±5 years), sex, residential district, and enrollment year. Finally, 100 sets of gastric cancer cases and matched controls were defined.

#### Extension phase

1) In December 2008, 95 new gastric cancer cases were additionally ascertained from the KMCC. These cases and 116 cases that were excluded in the discovery cohort due to prevalence status or inadequate DNA concentration were included in the extension phase (n = 211). Using the same matching method as the discovery phase, 211 controls were selected. 2) Gastric cancer cases were obtained from two university hospitals in Korea that were Chungnam University Hospital and Hanyang University GURI Hospital. From March 2002 to September 2006, a total of 490 gastric cancer patients were newly diagnosed at the hospitals. Their epidemiological data and venous whole blood samples were collected at the time of diagnosis or prior to gastric cancer surgery. Among them, 189 cases with sufficient DNA samples and informed consents were also included in the extension set. Also, 189 community-based controls were matched by age (±5 years) and sex from the KMCC subjects enrolled after 2000.

### Ethics Statement

The study protocols for the KMCC study, the hospital-based study and the current nested case-control study were approved by the institutional review boards (IRB) of Seoul National University Hospital and the National Cancer Center of Korea (H-0110-084-002 and C-0603-161-170) and by the institutional review board of Hanyang University Hospital (IRB no. 2003-4).

### Gene and SNP selection

Through literature review, we indentified 30 candidate genes that may be involved in the CagA signal transduction pathway linked to ERK downstream signaling by direct interaction with CagA or secondary affection in CagA genetic sequences [3], [7], [8], [9], [10], [11], [12]. The 30 candidate genes are as follows: v-Crk sarcoma virus CT10 oncogene homolog (*CRK*); v-Crk sarcoma virus CT10 oncogene homolog (avian)-like (*CRKL*); c-Src tyrosine kinase (*CSK*); growth factor receptor-bound protein 2 (*GRB2*); Met proto-oncogene (c-*MET*); nuclear factor of activated T-cells, cytoplasmic, calcineurin-dependent 4 (*NFATC4*); protein tyrosine phosphatase, non-receptor type 11 (*PTPN11*); spermine synthase (*SMS*); son of sevenless homolog 1(*SOS1*); v-Src sarcoma (Schmidt-Ruppin A-2) viral oncogene homolog (*SRC*); elk-related tyrosine kinase (*ERK*); focal adhesion kinase 1 (*FAK*); phospholipase C-gamma (*PLCγ*); v-Ki-ras2 Kirsten rat sarcoma viral oncogene homolog (*KRAS*); neuroblastoma RAS viral (v-ras) oncogene homolog (*NRAS*); v-raf murine sarcoma viral oncogene homolog B1 (*BRAF*); v-raf-1 murine leukemia viral oncogene homolog 1 (*RAF1*); mitogen-activated protein kinase kinase 1 (*MAP2K1*); mitogen-activated protein kinase kinase 3 (*MAP2K3*); mitogen-activated protein kinase kinase 4 (*MAP2K4*); mitogen-activated protein kinase kinase 5 (*MAP2K5*); mitogen-activated protein kinase kinase 6 (*MAP2K6*); cyclin-dependent kinase inhibitor 1A (*p21*); dedicator of cytokinesis 1 (*Dock180*); ras-related C3 botulinum toxin substrate 1 (*RAC1*); RAP1A member of RAS oncogene family (*RAP1*); WAS protein family, member 1 (*WAVE*); ARP2 actin-related protein 2 homolog (*Arp2*); ARP3 actin-related protein 3 homolog (*Arp3*); Rap guanine nucleotide exchange factor (GEF) 1 (*C3G*).

### Genotyping

#### Discovery phase

Genotyping was performed using the genome-wide human SNP Array 5.0 according to the standard protocol recommended by the manufacturer's instructions [15]. Before genotyping, concentrations of genomic DNA for all study subjects were measured using a spectrophotometer (NanoDrop ND-1000, NanoDrop Technologies). For each individual assayed, 250 ng of genomic DNA was digested with a restriction enzyme (Nsp I or Sty I). Though we screened a total of 580 SNPs within +/−5 kbp of the 30 target gene locations, 103 polymorphisms were excluded due to a SNP call rate of less than 95% or a HWE value less than 0.0001. Because the genome-wide human SNP Array 5.0 was manufactured based on a Caucasian population, 115 SNPs did not meet the criteria of MAF<0.05 in Asians and were also excluded. Additionally, 20 cases and 14 controls were excluded due to insufficient genomic DNA (<250 ng), sex discordance or poor genotyping (<90%). Finally, 362 SNPs in 30 genes (genotyping rate of 99.5%) were genotyped in 81 cases and 85 controls. The cluster images of signal intensity were reviewed for all SNPs.

#### Extension phase

Seven SNPs with raw or permutated *p* value<0.02 (rs5999749, rs9418677, rs4635002, rs10901081, rs7853122, rs530801, rs747182) identified in the discovery analysis were genotyped using the Illumina VeraCode GoldenGate Assay with BeadXpress according to the manufacturer's protocol (Illumina, San Diego, CA, USA) [16]. To ensure the reliability of the two genotyping methods, 135 samples (59 cases and 76 controls) were genotyped twice by both the genome-wide human SNP Array 5.0 and the Illumina VeraCode GoldenGate Assay, and the concordance rate was >98.2%. Of the 7 SNPs, rs9418677 was excluded due to a SNP call rate <95%. Two cases and 40 controls with low DNA availability (n = 15) or genotyping call rate <90% (n = 27) were also excluded in the analysis. Finally, six SNPs in five genes (genotyping rate of 99.1%) were analyzed in 398 cases and 360 controls in the extension phase.

### *H. pylori* and CagA detection

*H. pylori* infection status and CagA seropositivity were evaluated using immunoblot assay, Helico Blot 2.1™ (MP Biomedicals Asia Pacific, Singapore). Helico Blot 2.1™ kits have reported a sensitivity of 99% for both *H.pylori* and CagA seropositivity and a specificity of 98% for *H. pylori* and 90% for CagA seropositivity [17].

### Statistical analysis

The Hardy-Weinberg equilibrium (HWE) in the control group was evaluated by Fisher's exact test with a cut-off level of HWE<0.0001.

In the primary scan in the discovery set, the association between individual SNPs and gastric cancer risk was evaluated based on both raw and permutated *p*-values using the LRT with 1 degree of freedom in the trend (additive) model. The trend test assumes a dose-response effect with an increasing number of variant alleles. Permutated *p*-values were estimated by 100,000 permutation tests. Based on the additive model, gastric cancer risk was calculated as odds ratios (ORs) and 95% confidence intervals (CIs) using unconditional logistic regression model adjusting for risk factors that were smoking status (ever *vs.* never), *H. pylori* infection (positive *vs.* negative) and CagA seropositivity (positive *vs.* negative). The Benjamini-Hochberg false discovery rate (BH-FDR) corrected *p*-values of each SNP was computed to avoid spurious association with false positive outcomes [18].

In the extension phase, the most significant SNPs with raw or permutated *p* value<0.02 identified in the discovery phase were re-evaluated. Based on the additive model, gastric cancer risk was estimated as ORs and 95% CIs using unconditional logistic regression model adjusting for risk factors that were smoking status (ever *vs.* never), *H. pylori* infection (positive *vs.* negative) and CagA seropositivity (positive *vs.* negative). To summarize the results from the discovery and the extension analyses, data-pooling and meta-analysis were conducted. The summary ORs and 95% CIs were calculated using a fixed-effect model and heterogeneity was evaluated by the Cochran Q statistics [19].

All statistical analyses were performed using SAS software version 9.1 (SAS Institute, Cary, North Carolina) and PLINK software version 1.06 (http://pngu.mgh.harvard.edu/purcell/plink) [20]. Meta-analyses were conducted using STATA version 10 (Stata, College Station, TX).

## Results

Table 1 summarizes basic characteristics of the study participants in each phase. Gastric cancer cases showed significantly higher rates of CagA seropositivity (*p* = 0.03 in discovery phase, *p* = 0.09 in extension phase and *p* = 0.02 among total subjects). *H. pylori* infection, VacA seropositivity and smoking status were also higher among gastric cancer cases (Table 1).

**Table 1:** Basic characteristics of the study populations: Discovery and extension sets of community controls and gastric cancer cases from the KMCC, Chungnam University Hospital and Hanyang University GURI Hospital.

		Discovery phase^a^			Extension phase^b^			Total gastric cancer cases *vs.* controls		
		Case(N = 81)	Control(N = 85)	*p*-value	Case(N = 398)	Control(N = 360)	*p*-value	Case(N = 479)	Control(N = 445)	*p*-value
**Age**	Mean (SD)	64.2 (±7.9)	63.3 (±8.0)	0.44	61.6 (±10.4)	63.2 (±8.3)	0.02	62.0 (±10.1)	63.2 (±8.2)	0.06
**Sex**	Female	26 (32.1)	26 (30.6)	0.83	131 (32.9)	115 (31.9)	0.78	157 (32.8)	141 (31.7)	0.72
***H.pylori*** ** infection**	Positive (+)	72 (88.9)	68 (80.0)	0.11	353 (88.7)	309 (85.8)	0.24	425 (88.7)	377 (84.7)	0.07
**CagA**	Positive (+)	78 (96.3)	74 (87.1)	0.03	366 (91.9)	318 (88.3)	0.09	444 (92.7)	392 (88.1)	0.02
**VacA**	Positive (+)	50 (61.7)	46 (54.1)	0.32	279 (70.1)	240 (66.7)	0.31	329 (68.7)	286 (64.3)	0.16
**Smoking status** c	Ever smokers	52 (64.2)	47 (55.3)	0.24	247 (62.1)	196 (54.4)	0.10	300 (62.6)	243 (54.6)	0.01
**Drink status** d	Ever drinkers	46 (56.8)	50 (58.8)	0.79	250 (62.8)	213 (59.2)	0.28	296 (61.9)	263 (59.1)	0.38
**Gastric ulcer history**	Positive (+)	14 (21.9)	9 (14.1)	0.25	62 (17.8)	53 (19.1)	0.69	76 (18.5)	62 (18.1)	0.91

a Incidence gastric cancer cases identified in December 2005 and their age-sex matched controls from the KMCC.

b Newly identified and prevalent gastric cancer cases from the KMCC and newly enrolled gastric cancer cases from Chungnam University Hospital and Hanyang University GURI Hospital and their age-sex matched controls from the KMCC.

c Ever smokers were defined as former and current smokers.

d Ever drinkers were defined as former and current drinkers.

In the primary scan of the discovery set, of the 362 SNPs selected from CagA-related genes in the signal transduction pathway, 24 SNPs in eight genes, ERK, Dock180, C3G, Rap1, Src, CrkL, Mek and Crk, were significantly associated with gastric cancer risk in the single SNP analysis (*p*<0.05). According to the 100,000 permutation test, ERK rs5999749 and Dock180 rs9418677 presented a stronger association with gastric cancer (*p*<0.01). These SNPs showed significant gene-dose effects in the linear trend test and were significantly associated with an increased risk of gastric cancer (OR = 2.83, [95% CI: 1.42–5.65] for ERK rs5999749; OR = 1.90, [95% CI: 1.18–3.05] for Dock180 rs9418677). Except for Dock180 rs9418677 and Rap1 rs17028287, most SNPs downstream from CagA-Crk signaling (Dock180, C3G, Rap1 and Mek) were significantly associated with a reduced risk of gastric cancer (Table 2).

**Table 2:** Significant SNPs for candidate genes involved in downstream signaling pathways activated by CagA associated with gastric cancer in the discovery phase.

GENE	db SNP ID	#SNPs^a^	MAF (%)^b^	CHR^c^	CHRposition	*Raw* *p-value* d *	*Permutated* *p-value* e	OR (95% CI)^f^
***ERK***	rs5999749^g^	13	C ( 9.4)	22	20517660	0.0012	0.0011	2.83 (1.42–5.65)
***Dock180***	rs9418677^g^^,^ h	103	C (40.5)	10	128695397	0.0052	0.0048	1.90 (1.18–3.05)
	rs4635002^g^		A (11.3)		128752669	0.0119	0.0168	0.44 (0.18–1.07)
	rs7068941		A (11.8)		128718078	0.0418	0.0667	0.41 (0.17–0.99)
	rs7917277		T (11.8)		128754664	0.0418	0.0667	0.41 (0.17–0.99)
	rs9418832		A (11.8)		128757891	0.0418	0.0667	0.41 (0.17–0.99)
	rs9418737		A (11.8)		128757840	0.0418	0.0667	0.41 (0.17–0.99)
***C3G***	rs10901081^g^	30	A (21.2)	9	133586496	0.0109	0.0103	0.49 (0.27–0.91)
	rs7853122^g^		G (17.1)		133570432	0.0199	0.0248	0.47 (0.24–0.94)
	rs4991743		A (17.1)		133597568	0.0199	0.0248	0.47 (0.24–0.94)
	rs7047157		C (17.1)		133503399	0.0199	0.0248	0.47 (0.24–0.94)
	rs4474069		T (17.5)		133501207	0.0248	0.0260	0.49 (0.25–0.98)
	rs1544305		C (16.5)		133480201	0.0355	0.0361	0.50 (0.25–1.02)
***Rap1***	rs530801^g^	16	A (34.5)	1	111968778	0.0184	0.0186	0.57 (0.35–0.94)
	rs558989		G (32.9)		112043318	0.0207	0.0209	0.57 (0.34–0.95)
	rs17028287		T ( 4.2)		112046321	0.0215	0.0238	2.46 (0.96–6.28)
	rs571020		G (33.9)		112002851	0.0244	0.0266	0.59 (0.37–0.96)
	rs846261		A (32.4)		112054920	0.0305	0.0378	0.60 (0.36–0.99)
***Src***	rs747182^g^	5	G (12.9)	20	35416303	0.0224	0.0188	1.80 (0.99–3.26)
***CrkL***	rs5761368	2	A ( 9.4)	22	19607471	0.0281	0.0344	2.00 (1.01–3.96)
***Mek***	rs4255740	6	T (35.3)	15	64559061	0.0432	0.0426	0.58 (0.35–0.98)
	rs16949924		C (35.3)		64514651	0.0432	0.0426	0.58 (0.35–0.98)
***Crk***	rs8064892	6	C (14.7)	17	1283132	0.0477	0.0602	0.51 (0.24–1.07)
	rs8073032		C (14.7)		1283157	0.0477	0.0602	0.51 (0.24–1.07)

a Total number of selected SNPs within each candidate gene.

b Minor allele frequency among controls.

c Chromosome number.

d Raw *p*-values calculated in the trend model with a cut-off level ≤0.01.

e 100,000 permutations for single SNP in the trend model.

f Adjusted for age, smoking (never *vs.* ever), *H. pylori* infection (positive *vs.* negative) and CagA seropositivity (positive *vs.* negative).

g Seven representative SNPs with a raw or permutated *p* value<0.02 identified in the discovery phase were analyzed in the extension phase.

h Excluded due to a SNP call rate <95%.

*All BH-FDR *p*-values were not significant (*p*>0.05).

In the extension phase, all associations between the selected SNPs and gastric cancer risk were relatively attenuated. ERK rs5999749, Dock180 rs4635002 and C3G rs7853122 showed significant gene-dose effect for gastric cancer (OR = 1.40, [95% CI: 1.04–1.89]; OR = 0.65, [95% CI: 0.44–0.94]; OR = 0.61, [95% CI: 0.46–0.81], respectively). In the final combined analysis that included the discovery and extension analyses, the risk estimates of ERK rs5999749 were significantly associated with gastric cancer in both the pooled analysis and meta-analysis (OR = 1.57, [95% CI: 1.20–2.07]; OR = 1.56, [95% CI: 1.19–2.06], respectively). Moreover, Dock180 rs4635002 and C3G rs7853122 showed significantly decreased risk of gastric cancer in both analyses (OR = 0.60, [95% CI: 0.42–0.84], OR = 0.61, [95% CI: 0.43–0.87] for Dock180 rs4635002; OR = 0.59, [95% CI: 0.45–0.77], OR = 0.59, [95% CI: 0.45–0.76] for C3G rs7853122). There was no heterogeneity across the studies (Cochran Q test, *p*>0.05) except for rs10901081 (*p* = 0.040) (Table 3).

**Table 3:** Association between representative SNPs and gastric cancer risk in 479 gastric cancer cases and 445 controls.

		Discovery phase^a^		Extension phase^b^		Total gastric cancer cases *vs.* controls		
GENE	SNP	MAF^c^ (%)	OR (95% CI)^d^	MAF^c^ (%)	OR (95% CI)^d^	MAF^c^ (%)	OR (95% CI)^d^^,^ e	OR (95% CI)^d^^,^ f *
***ERK***	rs5999749	C ( 9.8)	2.83 (1.42–5.65)	C (11.0)	1.40 (1.04–1.89)	C (10.5)	1.57 (1.20–2.07)	1.56 (1.19–2.06)
***Dock180***	rs4635002	A (11.3)	0.44 (0.18–1.07)	A (11.1)	0.65 (0.44–0.94)	A (11.1)	0.60 (0.42–0.84)	0.61 (0.43–0.87)
***C3G***	rs7853122	G (17.1)	0.47 (0.24–0.94)	G (18.2)	0.61 (0.46–0.81)	G (18.0)	0.59 (0.45–0.77)	0.59 (0.45–0.76)
	rs10901081	A (21.2)	0.49 (0.27–0.91)	A (18.2)	0.98 (0.75–1.27)	A (18.5)	0.87 (0.68–1.10)	0.88 (0.69–1.12)
***Rap1***	rs530801	A (34.5)	0.57 (0.35–0.94)	A (25.8)	0.93 (0.73–1.19)	A (27.5)	0.85 (0.68–1.05)	0.85 (0.68–1.05)
***Src***	rs747182	G (12.9)	1.80 (0.99–3.26)	G (16.7)	1.05 (0.80–1.39)	G (16.0)	1.17 (0.92–1.50)	1.16 (0.90–1.48)

a Incidence gastric cancer case identified in December 2005 and their age-sex matched controls from the KMCC.

b Newly identified and prevalent gastric cancer cases from the KMCC and newly enrolled gastric cancer cases from Chungnam University Hospital and Hanyang University GURI Hospital and their age-sex matched controls from the KMCC.

c Minor allele frequency among controls.

d Adjusted for age, smoking (never *vs.* ever), *H*. *pylori* infection (positive *vs.* negative)and CagA seropositivity (positive *vs.* negative).

e Pooled analysis including all gastric cases and controls form each study dataset.

f Meta analysis using fixed effect model for combined analysis.

*No heterogeneity across the phases (Cochran Q test, *P*-heterogeneity >0.05) except for rs10901081 (*p* = 0.040).

## Discussion

In our multi-stage genetic analysis, three SNPs, ERK rs5999749, Dock180 rs4635002 and C3G rs7853122, showed strong associations with gastric cancer and may be important regulatory factors in the CagA signal transduction pathway.

Extracellular signal-regulated kinase (ERK), also known as mitogen-activated protein kinase (MAPK), is an important integration point for multiple cellular signals that regulates various oncogenic responses. The ERK signal pathway interacts with a considerable number of substrates, including protein kinases, phosphatases, cytoskeletal components, apoptosis regulators, and a range of other signaling-related molecules [21]. CagA signaling is one of the streams involved in the ERK signal cascade. Numerous studies have reported that CagA-positive *H. pylori* can activate ERK in gastric epithelial cells to promote inappropriate cellular functions [11], [21], [22], [23]. Moreover, interaction between CagA and signal transduction proteins promotes ERK signaling in conjunction with Ras, Mek and NF-kB, inducing gastric carcinogenesis. In cellular mechanisms, ERK appears to be involved in a wide variety of cellular processes. Thus, the host gene of ERK protein may be more important in determining protein expression and capacity. The results of this study demonstrate that ERK rs5999749 is primarily selected in SNP-based analysis and retains its strong association with gastric cancer in the final combined analyses. This supports that its genetic effect can play a critical role in gastric carcinogenesis equal to its protein activity level at the cellular stage.

Dock180, synonymous with a dedicator of cytokinesis 1, is a 180 kDa protein downstream-combining molecule of Crk and up-regulator of Rac1 [24]. It modulates various functions, including cell spreading, cell migration, and actin cytoskeletal organization through activation of Rac1 [24], [25], [26], [27], [28]. This protein is one of the Crk-downstream proteins involved in the cascade of CagA and Crk signaling through the Crk-Codk180-ELMo pathway [9]. Similarly, C3G known as Rap guanine nucleotide exchange factor (GEF) 1 (RAPGEF1) also interacts with the Crk [29]. Previous studies demonstrated that the Crk-C3G-Rap1 signaling can activate the ERK cascade and induce apoptosis, cell growth, migration, adhesion and mortality [30], [31], [32]. In human carcinogenesis, the C3G gene appears to play a crucial role by itself. Alteration of the C3G genetic activity via amplification or methylation is associated with several cancers such as lung, gastrointestinal and gynecological cancers [33], [34]. Although it is not exactly well known whether the genetic variants of the Dock180 and C3G gene are linked to gastric carcinogenesis, our study suggests several SNPs in these genes, especially rs4635002 and rs7853122, are significantly associated with risk of gastric cancer, and thus may be a susceptible gene in the development of gastric cancer.

**Figure 1:**
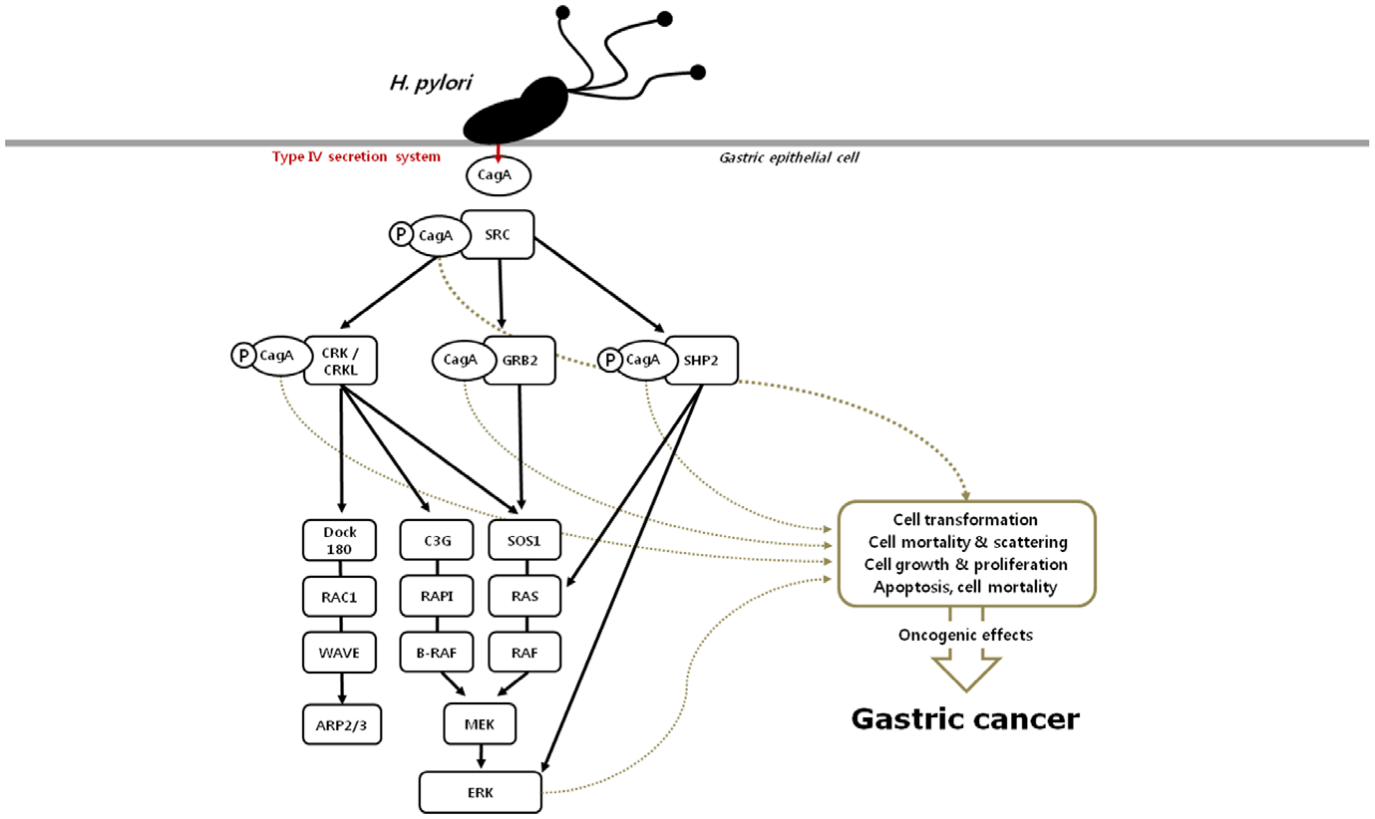
CagA oncogenic effect via the ERK signal pathway. The CagA interaction can stimulate the downstream signals in the ERK cascade linked to aberrant cellular functions that leads to gastric carcinogenesis. In this process, the genetic effects of ERK, Dock180 and C3G play critical roles equal to their protein activity levels at the cellular stage.

Based on the present results and review of cellular mechanisms [3], [9], [11], [21], [22], [23], [24], [30], CagA oncogenicity induced by activation of the ERK signal pathway can be infered (Figure 1). The CagA interaction with binding molecules such as Src, Crk, GRB2 and SHP-2 stimulates the downstream signals in the ERK cascade linked to aberrant cellular functions that leads to gastric carcinogenesis. During this process, the genetic effects of ERK, Dock180 and C3G can play critical roles equal to their protein activities. These results provide support for the genetic and cellular importance of those molecules.

Our genetic analysis presented plausible evidence on genetic variants of the ERK signal transduction pathway activated by CagA, but several limitations should be noted. First, the number of study subjects was insufficient to ensure statistical power to assess the exact association between selected SNPs and gastric cancer risk. Second, due to the small sample size and lack of cardiac gastric cancer patients (less than 5%), we could not conduct stratified analysis according to gastric cancer type, cardiac *vs.* non-cardiac. Therefore, results should be interpreted with caution.

This study indicates that genes involved in the ERK signal transduction pathway activated by CagA can modify risk of gastric cancer. ERK, Dock180 and C3G genes may play important roles in the development of gastric cancer. Replication studies in other populations will allow us to elucidate gastric cancer pathological mechanisms. Further biological studies focused on these genes can clarify their roles in gastric carcinogenesis.
